# Thyroglossal Duct Cyst Carcinoma With Synchronous Thyroid Papillary Carcinoma: A Case Report and Literature Review

**DOI:** 10.7759/cureus.28570

**Published:** 2022-08-30

**Authors:** Carla Peres, Nuno Rombo, Leonor Guia Lopes, César Simões, Rita Roque

**Affiliations:** 1 Surgery and Renal Transplantation, Hospital de Santa Cruz - Centro Hospitalar de Lisboa Ocidental, Lisbon, PRT; 2 Endocrinology, Hospital Egas Moniz - Centro Hospitalar de Lisboa Ocidental, Lisbon, PRT

**Keywords:** sistrunk’s procedure, papillary carcinoma, thyroid carcinoma, thyroglossal carcinoma, thyroglossal duct cyst

## Abstract

Thyroglossal duct cysts (TGDC) are one of the most common congenital anomalies in the neck. Malignant transformation of these cysts is rare and synchronous involvement of the thyroid gland is even rarer. We report a case of synchronous occurrence of carcinoma in the thyroglossal duct cyst and thyroid gland and review the relevant literature. A 24-year-old woman who presented with a midline cervical mass, clinical examination, and complementary study was suggestive of a thyroglossal cyst with papillary carcinoma on fine-needle aspiration biopsy (FNAB) synchronous with thyroid papillary carcinoma with no cervical ganglion metastases documentation. Sistrunk’s procedure plus total thyroidectomy was performed. With the clinical resemblance of benign and malignant cysts and the limitations of imaging techniques to distinguish between them, FNAB might be of use. Surgical treatment is warranted for the treatment of thyroglossal duct cyst carcinoma, but controversy still exists as to the extent of the surgical intervention. Sistrunk’s procedure seems to be considered the gold standard when there is no evidence of thyroid involvement. However, in the presence of concomitant thyroid carcinoma, total thyroidectomy and cervical lymphadenectomy for evident node metastases are required. In the case of synchronous thyroglossal and thyroid carcinoma, most authors recommend pursuing both radioiodine therapy and hormone ablation. Thyroglossal duct cyst carcinoma is an uncommon feature that can arise from clusters of thyroid cells found within the cyst as in the present case. When confronted with this diagnosis it is fundamental to take into consideration the possibility of synchronous lesions as well as the extent of local and distance disease, since it has a direct influence on the choice of treatment provided to the patient. As there was a synchronous presence of papillary carcinoma in both the thyroglossal cyst and the thyroid gland, both the Sistrunk procedure and total thyroidectomy were performed, and radioiodine therapy was pursued as well as hormone ablation.

## Introduction

Thyroglossal duct cysts (TGDC) are the most common congenital anomalies in the neck, accounting for about 7% of midline cervical masses in the adult population. Malignant transformation of these cysts is rare [1-3]. Since its first description by Brentano in 1911, there have only been nearly 277 cases described in the literature. Synchronous involvement of the thyroid gland is even rarer [2,4,5]. We report a case of synchronous occurrence of carcinoma in the TGDC and thyroid gland and review the relevant literature.

## Case presentation

**Figure 1:**
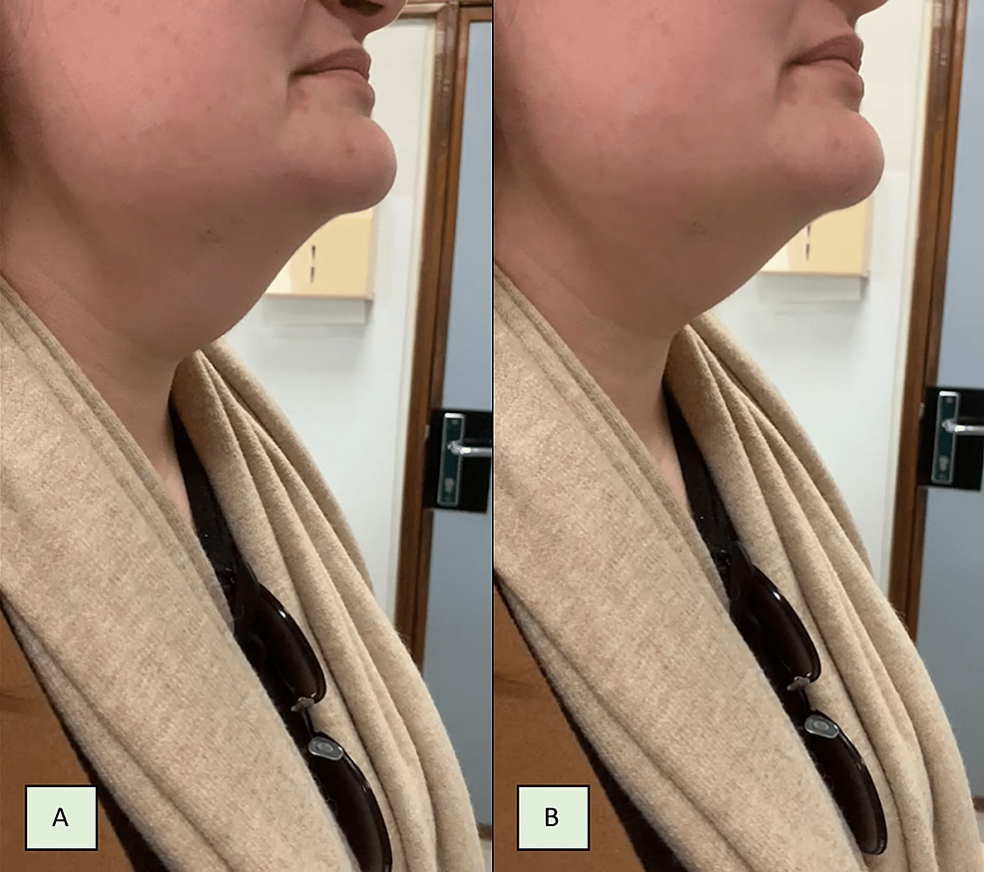
The patient’s clinical presentation of midline cervical mass mobile with deglutition A: Rest position, B: Deglutition

We present a case of an otherwise healthy 24-year-old woman with a six-month history of an anterior cervical mass. She had no other associated complaints. The patient had smoking habits (2.25 pack years) and had a positive family history of benign thyroid pathology. On physical observation she presented with an antero-superior central cervical mass of firm but not petrous consistency, mobile with both deglutition and tongue protrusion, suggestive of a thyroglossal cyst; the thyroid was not palpable and there were no palpable cervical lymph nodes (Figure 1).

The patient had normal thyroid function tests (thyroid-stimulating hormone (TSH), triiodothyronine (T3), and free thyroxine (fT4) serum levels), normal calcitonin levels (<0,5pg/mL), and serum thyroglobulin level >500ng/mL.

**Figure 2:**
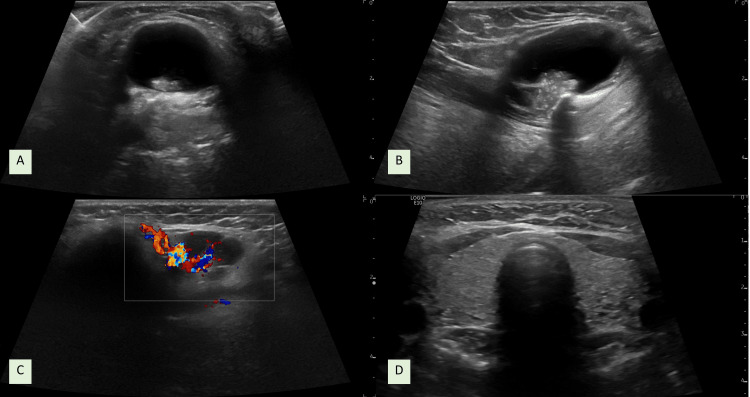
First cervical ultrasonography A and B: Thyroglossal duct cyst transversal and longitudinal views, C: Thyroglossal duct cyst showing solid component with positive Doppler sign, D: Thyroid gland transversal view

Imaging studies with cervical ultrasonography showed a lobular mass of 2.7x3.9cm by the thyroglossal duct and thyroid micronodularity without clinical significance (Figure 2). A fine needle aspiration biopsy (FNAB) on this cervical mass was conclusive of papillary thyroid carcinoma. Based on this finding, thyroid ultrasonography was repeated, showing the presence of an oval 7x6x8mm solid nodular image with ill-defined limits and micro calcifications, on the right side as per European Thyroid Association's Thyroid Imaging Reporting and Data Systems (EU-TIRADS 5) (American College of Radiology (ACR) TI-RADS TR4 - 6 points). The FNAB of this lesion was positive for the presence of papillary carcinoma (Bethesda, malignant).

**Figure 3:**
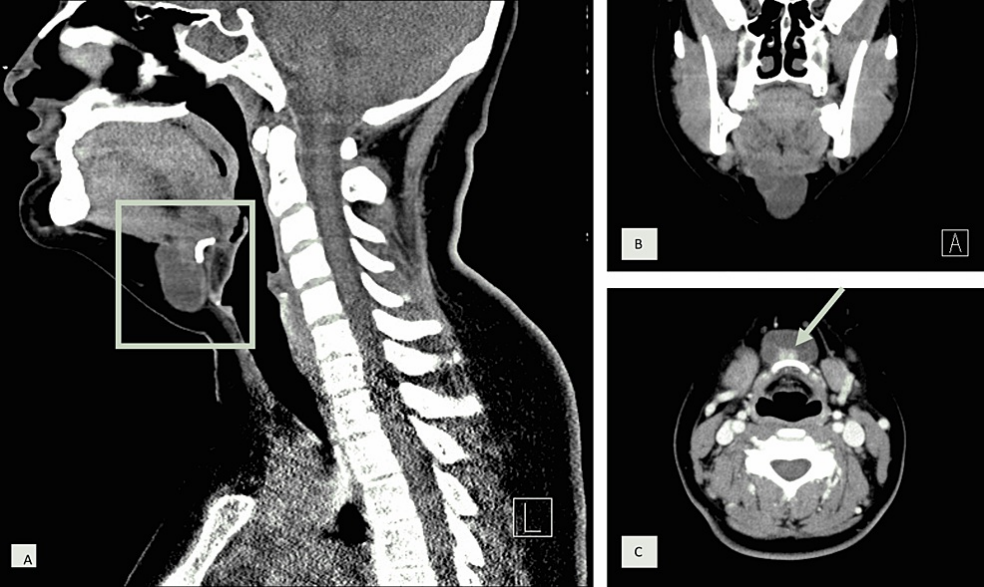
Cervical CT-angiogram A: Sagittal plane revealing cystic mass adjacent to the hyoid bone (rectangle), B: Coronal plane with the thyroglossal cyst extending to the base of the tongue, C: Axial plane showing cystic multiseptated mass anterior to the hyoid bone; solid intracystic component (arrow)

The investigation proceeded with a cervical CT-angiogram, revealing a midline cystic multiseptated lobular lesion of 31x17x31 mm that correlated with the thyroglossal duct, and with a solid component anterior to the hyoid bone. This intracystic solid component measured 10x7x14 mm, was spontaneously dense, and had contrast enhancement, probably corresponding to ectopic thyroid tissue with focal papillary carcinoma (Figure 3). This mass was adherent but did not invade the hyoid bone which showed cortical integrity, and had an intramuscular component in the base of the tongue. There were no suspicious cervical lymph nodes.

On cervical ultrasonography, there were no pathological findings on the latero-cervical lymph node chains and only one oversized submandibular lymph node was noted. Although this submandibular node had no imageological characteristics suggestive of malignancy, FNAB was performed to exclude metastases. Histopathology revealed the sample's cellularity was insufficient for diagnosis and thyroglobulin measurement was almost untraceable (0.12 ng/mL).

**Figure 4:**
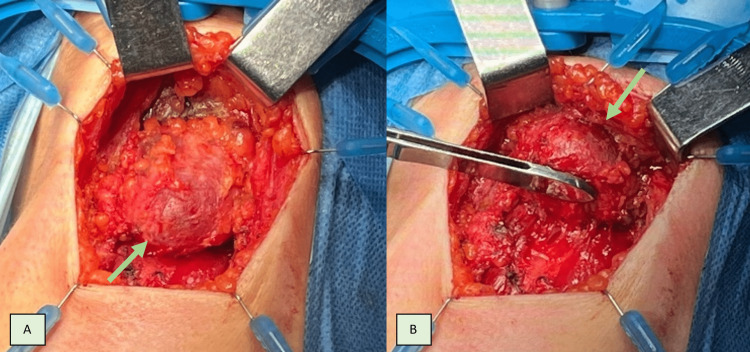
Transversal skin incision was made according to Langer’s lines at the level of the hyoid bone, followed by individualization of the thyroglossal cyst (arrow) until the hyoid bone. A: Anterior dissection, B: Posterior dissection

**Figure 5:**
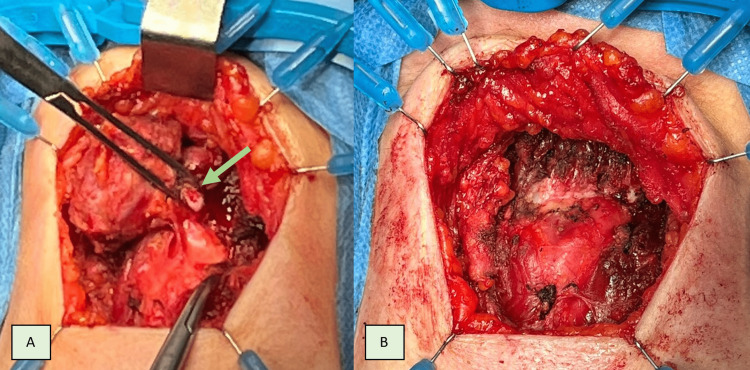
Sistrunk’s procedure A: The mid-portion of the hyoid bone with the sectioned hyoid bone demarcated by the arrow, B: Completion of Sistrunk’s procedure

**Figure 6:**
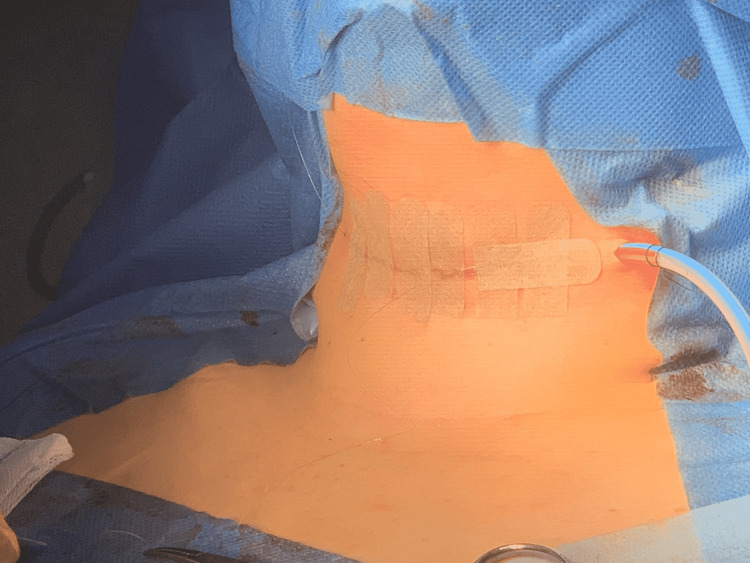
Closure with 15mm Blake aspirative drain left on the loci of total thyroidectomy

**Figure 7:**
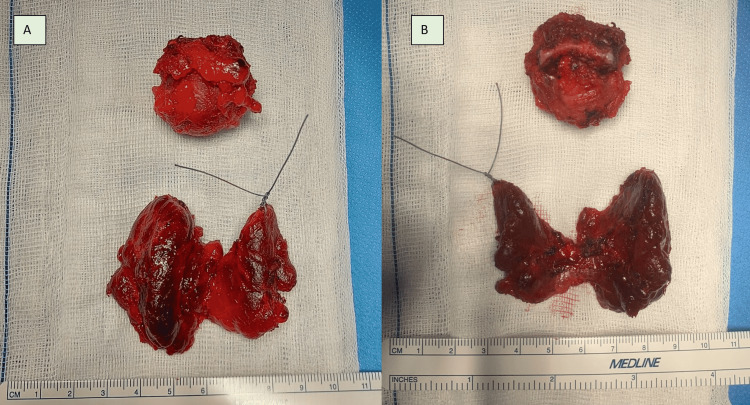
Pathological specimens of Sistrunk's procedure (superior) and of total thyroidectomy. The left superior lobe is marked with suture (inferior). A: Anterior view, B: Posterior view

The patient was then discussed in a multidisciplinary meeting and proposed for surgical treatment. Sistrunk’s procedure plus total thyroidectomy without cervical lymph node dissection were executed (Figures 4-7).

The postoperative period occurred without complications. The cervical drain was removed on the first postoperative day and the patient was discharged on levothyroxine.

Histopathology findings of the surgical specimens revealed a multifocal classical papillary carcinoma of the thyroid and the presence of thyroid tissue within the thyroglossal cyst with papillary carcinoma presenting with local invasion of the surrounding soft tissue and hyoid bone.

After a new multidisciplinary meeting, the patient was proposed to continue treatment with radioactive iodine.

## Discussion

Thyroglossal duct cysts are formed when there is a failure of closure of the thyroglossal duct between the eighth and 10th week of gestation, and the epithelial remnants are reorganized into a small cervical mass [1]. These account for nearly 7% of midline cervical masses in the adult population [2] and are the commonest congenital anomalies in the neck [3]. The TGDC has a very low potential for malignant transformation with only 0.7% to 1.5% of cases described to present as carcinomas. They most commonly occur in the fourth decade of life [4] and are more prevalent in women [5]. Since its first description by Brentano in 1911, there have only been nearly 277 cases described in the literature [2].

The etiology of thyroglossal duct cyst carcinoma is still debated. Some claim that these carcinomas arise from clusters of normal thyroid cells found in these cysts. Another theory postulates these can be metastases from an occult primary tumor [2]. The histopathology of these tumors in decreasing order of frequency is papillary carcinoma (80%) followed by ‘mixed’ papillary-follicular carcinoma (8%) and squamous cell carcinoma (6%). Hurthle cell, follicular and anaplastic carcinoma comprise the remaining 6%, while medullary carcinoma of the TGDC has not been reported [6]. Primary papillary carcinoma in TGDC presents with neck node metastases reported in 7.7% to 12.9% of cases and it is rare to find distant metastases [5]. Synchronous papillary carcinoma of the thyroglossal duct cyst and papillary thyroid carcinoma occur in 11% to 33% of cases and are probably associated with a multifocal pattern of presentation rather than a metastatic disease [5].

The clinical pattern is the same for benign cysts with the patient presenting an anterior cervical mass usually without any other symptoms. High suspicion should be raised by a rapid rate of growth, and the presence of a hard, fixed, and/or irregularly shaped mass [2]. Our patient's cervical mass had a six-month period of slow progressive growth.

Imaging techniques are not able to distinguish benign from malignant tumors [7]. The FNAB only yields a correct result in approximately 50% to 66% of cases [8]. When there is a positive biopsy, the thyroid gland should be carefully studied to rule out thyroid concomitant pathology. A definitive diagnosis is based on pathological findings of the surgical specimen [1].

Surgical treatment is warranted for the treatment of TGDC carcinoma. Surgical excision of the cyst alone is an inadequate procedure as it renders a high recurrence rate (50%). The adoption of the Sistrunk procedure i.e., the removal of the thyroglossal duct cyst, the medial segment of the hyoid bone, and a core of tissue around the duct to open into the oral cavity at the foramen cecum [1,9,10], can reduce this recurrence rate to as low as 3% [6].

A revision of the previously established Sistrunk technique has since been described. This procedure encompasses a suture-guided transhyoid pharyngotomy, allowing better management of the difficulties encountered when attempting complete removal of all epithelial remnants of the thyroglossal duct in recurrent cases, as it gives direct access to the tissues between the hyoid bone and the foramen cecum that can be excised under direct vision while avoiding injury to vascular nervous structures [11]. Even though the concept might be promising there are still only a few cases described to date and there is no sufficient data to recommend this technique over Sistrunk’s procedure.

There is some controversy regarding the role of total thyroidectomy as the initial treatment for these patients. Some believe these tumors to be metastatic thyroid disease and therefore advocate for initial simultaneous total thyroidectomy with the Sistrunk procedure [12,13]. For those who believe this to be a primary disease of the thyroglossal duct cyst, in the absence of thyroid pathological findings in the diagnostic evaluation, there is no need for total thyroidectomy as an initial procedure [12]. Risk-stratification of the patient prior to the surgical procedure can also help in the decision-making process as it is believed that for patients who yield a low risk (aged under 45 years, no previous history of radiation exposure, low-grade tumors without cyst wall invasion, no evidence of lymph node or distant metastases) total thyroidectomy should not be performed, and a long-time period of observation should be held [5]. Recommendations for total thyroidectomy along a Sistrunk’s procedure as initial management are devised for patients who present features of high risk: thyroid gland is found to be nodular with suspicion on ultrasonography or a cold nodule in a thyroid scan; presence of clinically/sonographically detected lymphadenopathy; or in the presence of the previous history of neck irradiation [6].

The incidence of reported cervical lymph node metastasis from thyroglossal carcinoma is lower than what is seen with papillary carcinoma of the thyroid gland, and death from this disease is rare [14]. Accordingly, cervical lymphadenectomy is only recommended if there is suspected lymph node invasion. In that case, besides Sistrunk’s procedure concomitant total thyroidectomy and, following the same criteria as for thyroid cancer, appropriate level selective neck dissection should be performed [14,15]. In some small series, there has been no significant outcome difference in long-term survival between patients who required lymphadenectomy as part of the initial procedure and those who did not require it [16]. However, these reports are gathered from trials based on small clusters.

Another controversial issue is the role of postoperative hormone suppression and/or ablation with radioactive iodine. Most authors who recommend total thyroidectomy are in favor of postoperative radioactive iodine ablation and levothyroxine therapy at a suppressive dose with periodic whole-body scintigraphy during follow-up [1]. However, a comparative retrospective review of Patel et al. showed a statistically significant difference in 10-year overall survival between patients receiving radioiodine therapy and the ones who did not, with survival rates of 50% and 100%, respectively. Nevertheless, we must keep in mind biased group selection with the patients receiving radioiodine therapy naturally belonging to a higher risk group [14,16].

As to the present, there is no significant data to establish guidelines regarding the matter of thyroid suppression when the Sistrunk procedure alone is performed, however, most experts believe in the beneficial effect of the suppression of one possible thyroid stimulus and therefore recommend treatment with thyroid-stimulating hormone between 0.1 and 0.5mIU/L [1, 14]. In those cases, follow-up is recommended to be done with annual thyroid stimulating hormone examination and thyroid gland ultrasound. Neither serum thyroglobulin measurement nor any other complementary studies with radioactive iodine are indicated [14].

In this case, the complementary investigation after the diagnosis of the thyroglossal cyst revealed the presence of synchronous thyroid papillary carcinoma. Therefore, a concomitant total thyroidectomy was performed. Since the staging was negative for either regional lymph nodes or distant metastases, no further dissection was considered necessary. However, as there was a synchronous papillary carcinoma in both the thyroglossal cyst and the thyroid gland, complementary radioiodine therapy after surgery was considered from the pre-operative period, as well as hormone ablation with levothyroxine. Moreover, the invasive features of the histopathology of the surgical specimens further support this strategy.

Regarding the prognostic factors, in a univariate analysis of overall survival predictors in patients with thyroglossal carcinoma, Patel et al. demonstrated that only the extent of surgery for the thyroglossal cyst was a significant outcome predictor, with a 10-year overall survival rate of 75% and 100% for the patients being subjected to simple cyst removal versus Sistrunk’s procedure [16,17]. The overall prognosis of patients with thyroglossal carcinoma is good and most cases are adequately treated by resection with the Sistrunk procedure, with a reported cure rate of 95% [14].

## Conclusions

Thyroglossal duct cysts are important to consider in the differential diagnosis of cervical masses as they are one of the most common congenital abnormalities in the neck. Thyroglossal duct cyst carcinoma (their malignant transformation) is a very rare condition and can arise from metastasis from distant carcinomas or clusters of thyroid cells found within the cyst as in the case of our patient.

Although it is an uncommon feature, when confronted with this diagnosis the surgeon must take into consideration the possibility of synchronous lesions (namely in the thyroid gland) as well as the extent of local and distance disease, and if necessary, repeat cervical imaging (which in the present report allowed the identification of suspicious thyroid nodules) and restage the disease, since it has a direct influence in the choice of treatment provided to the patient.

The disease has a favorable prognosis when adequately treated with a Sistrunk procedure reaching a very high cure rate. Nevertheless, it is of paramount importance to consider the diagnosis of papillary carcinoma with invasive features in the present case report which considerably worsens the prognosis even when complementary radioiodine plus hormone ablation therapy is pursued.
